# Knowledge, Attitude, and Practice of Vaccination Among Parents in Jeddah City, Saudi Arabia

**DOI:** 10.7759/cureus.41721

**Published:** 2023-07-11

**Authors:** Amany Ali Alghamdi, Hani A Alghamdi

**Affiliations:** 1 Preventive Medicine Postgraduate Program, Ministry of Health, Jeddah, SAU; 2 Preventive Medicine Department, Public Health Directorate, Ministry of Health, Jeddah, SAU

**Keywords:** myths, misconceptions, attitudes, awareness, mothers, caregivers, children, vaccination

## Abstract

Background

Vaccine misconception plays an important role in delaying vaccination for children, which can lead to serious health problems. Assessing the knowledge, attitude, and practice (KAP) and potential associated factors among Saudi parents of preschool and school-age children and adolescents for all types of vaccines would help increase vaccine coverage.

Methodology

This cross-sectional, questionnaire-based survey was performed among parents in Jeddah, Saudi Arabia. The study enrolled all Saudi parents visiting primary healthcare centers (PHC) in Jeddah with their children aged 3-18 years old. A self-administered questionnaire in Arabic was used to assess KAP. Descriptive statistics were performed, and the chi-square test was used to assess the association between KAP and sociodemographic factors with significance set at p-values <0.05.

Results

Out of 301 participants, 68.1% were mothers, and 55.8% of children were female. The largest percentage (81.7%) of the parents were aged between 31 and 50 years old. Although 94.4% of the parents agreed or strongly agreed that childhood vaccines protect their children from serious diseases, 26.6% of parents agreed or strongly agreed that vaccines can potentially cause autism, and 18.6% believed they could lead to learning difficulties. Most parents (67.4%) were in favor of vaccination, while others were hesitant about vaccinating their children and believed in alternative methods of disease prevention. About one-third knew other parents who did not vaccinate their children for religious, ideological, or cultural reasons. Sociodemographic factors such as gender (p = 0.042), educational level (p = 0.017), nationality (p = 0.001), and first child (p = 0.013) had some influence on parents’ beliefs and knowledge about vaccination, while the number of children (child order) (p = 0.022) and parents’ education level were associated with hesitancy (p = 0.028).

Conclusions

These findings show that most parents had good KAP toward vaccination, influenced by sociodemographic factors. However, there is a need to address vaccination hesitancy by acting on identified contributing factors.

## Introduction

Multiple outbreaks of diseases that can be prevented by vaccination are on the rise, putting children’s health at risk [1]. The declining vaccination rates and the burgeoning anti-vaccination efforts are two important contributing factors [2]. The anti-measles campaign, which started in 1998 following a false study tying the measles, mumps, and rubella (MMR) vaccine to autism [3], is perhaps the most well-known instance. Despite the discrediting of the original paper and its retraction, this error continues to cause worry and concern for many parents. Doctors continue to deal with hesitant families that contest the value of immunizations regularly. The anti-vaccination movement has been spreading to middle-income nations and a large number of Arab nations [4]. Although influenza infection is linked to severe morbidity and death, especially in young children, influenza vaccination is one of the vaccines that generate the most debate among parents. The WHO estimates that the influenza epidemic causes 3-5 million cases of severe illness with secondary complications each year and 290,000 to 650,000 reported global deaths, primarily affecting elderly people, patients with other comorbidities, and young children [5]. Although influenza vaccination is known to be the most efficient method of preventing influenza infection and reduces overall healthcare costs, coverage rates remain insufficient globally [6].

A study conducted by Almutairi et al. [7] showed most Saudi parents (87.2%) had good knowledge, attitude, and practice (KAP) toward immunization which was associated with female gender and higher education. Moreover, a cross-sectional study on parents from the Hail region of Saudi Arabia revealed that even though 18.4% of children had vaccination-related minor side effects during or after vaccination, of which 23.2% required doctor visits, 60-90% of respondents were aware of the health benefits of vaccinations for children [8]. On the other hand, a study by Alshammari et al. that assessed the level of knowledge about vaccination among Saudi parents showed that 73.3% of parents reported having a good understanding of childhood immunization, and 9% were aware that regular vaccinations shield kids from infectious diseases and associated repercussions [9]. There is no detailed information on the level of KAP toward influenza vaccines among parents in Saudi Arabia and the relationship between knowledge and psychological and personal characteristics. Bridging this lack of information might inform the establishment of successful intervention programs to increase vaccine coverage. Increasing awareness about the influenza vaccine and its correlates among parents of school-age children who spent most of their time together in classrooms would increase vaccine coverage and reduce infection among children who are particularly vulnerable to flu. Children under the age of five years, particularly those under the age of two years, are at a higher risk of developing significant flu-related complications [10,11]. According to the CDC, flu-related hospitalizations among children under five in the United States ranged from 6,000 to 27,000 per year between 2010 and 2020 [10]. Therefore, this study aimed to assess KAP toward influenza vaccine and potential associated factors among Saudi parents of preschool and school-age children and adolescents.

## Materials and methods

Study design and population

This is a cross-sectional, questionnaire-based survey study conducted from January 20 to June 5, 2023, among parents in Jeddah, Saudi Arabia. The study enrolled all parents visiting primary healthcare centers (PHCs) in Jeddah city with their children aged 3-18 years old (preschool, school, and adolescent age groups). Parents who refused to provide informed consent and caretakers who were not children’s parents were excluded.

Sample size and sampling

We calculated the sample size using Raosoft.com. Considering a prevalence of good knowledge reported in the literature to be between 25% and 30%, the minimal sample size to achieve a 95% confidence interval with a margin of error of 5% was about 300 participants.

During sampling, we used a multistage stratified sampling technique. In the first stage, Jeddah was divided into five sectors according to geographic location. In the second stage, in each sector, one PHC was selected randomly. In the third stage, we selected 77 subjects from each PHC using a systematic random sampling approach for a total of 385 participants.

Data collection tool

We used a pre-validated questionnaire from a study by Zakhour et al. [12], consisting of 50 questions in Arabic, with the following four sections: knowledge, attitude, and practice regarding childhood vaccines and sociodemographic data. For each of the knowledge and attitude sections, questions were grouped into themes. Three themes were identified for knowledge, namely, efficacy, safety, and general knowledge. For attitudes, the following three themes were identified: reasons for vaccination, trust, and hesitancy. Three other questions assessed the practice, asking about whether or not their children were vaccinated and the reasons. Responses to questions were measured on a five-point Likert scale. Further details on the questionnaire are in the study by Zakhour et al. [12]. The questionnaire was first tested among 30 parents from various socioeconomic backgrounds to assess its clarity, comprehension, length, and cultural appropriateness. Pilot test results were used to improve the questionnaire and were not included in the main study. The calculated Cronbach’s alpha was 0.84 showing good reliability.

The selected participants were invited to participate, and the questionnaire was distributed using a link to Google Forms through their smartphones. Those who did not have internet access were offered a device with internet connectivity to access the questionnaire.

Statistical analysis

Data were analyzed using SPSS software version 26 (IBM Corp., Armonk, NY, USA). Categorical variables were reported as absolute and relative frequencies and continuous variables were described as means and standard deviations (SDs). The associations were assessed using the chi-square test at a statistical significance of p < 0.05.

Ethical considerations

Approval to conduct the study was obtained from the Institutional Review Board Committee at the Ministry of Health.

## Results

A total of 301 respondents were included out of 385, corresponding to a response rate of 78%. Of all respondents, 55.8% were parents of female children, while 44.2% were parents of male children. Most were mothers (68.1%), while the rest were fathers (31.9%). Regarding the educational level of the children, the majority (51.5%) were in kindergarten, followed by 35.5% in primary school. Notably, only a small proportion (6.6% and 6.3%) of children were in intermediate and secondary school, respectively. Most (62.5%) were the oldest child in their families, while the remaining 37.5% were other siblings. The vast majority of parents surveyed were of Saudi nationality, constituting 95.7% of the sample, with a minority (4.3%) being non-Saudi. When examining the age distribution of parents, the largest percentage (81.7%) were within the 31-50-year age range, followed by 15.9% in the 21-30-year age range. The remaining parents were evenly split between the 18-20-year and above 50-year age groups, each representing 1.3% and 1.0%, respectively (Table 1). Regarding parents’ occupations, the majority (83.1%) were employed, whereas only a small percentage (3.3%) were engaged in private business, and 13.6% were unemployed. When considering the parents’ highest educational level, the majority (91.0%) held a university degree, followed by 7.3% with a secondary school education level. Only a minimal percentage (1.7%) reported having other forms of education. Finally, family income analysis revealed that 61.2% of families had a monthly income exceeding 18,750 Saudi Rials (SAR), while 38.8% had an income below that threshold (Table 1).

**Table 1:** Sociodemographic characteristics of the parents and children. SAR = Saudi Rial

Characteristics	Frequency	Percent (%)
Gender of the child		
Male	133	44.2
Female	168	55.8
Educational level of the child		
kindergarten	155	51.5
Primary school	107	35.5
Intermediate school	20	6.6
Secondary school	19	6.3
Child order		
The oldest	188	62.5
Other	113	37.5
Nationality		
Saudi	288	95.7
Non-Saudi	13	4.3
Surveyed parent		
Mother	205	68.1
Father	96	31.9
Age of the parent (years)		
18–20	4	1.3
21–30	48	15.9
31–50	246	81.7
>50	3	1.0
Occupation of the parent		
Employee	250	83.1
Private business	10	3.3
Unemployed	41	13.6
Highest educational level of the parent		
Secondary school	22	7.3
University degree	274	91.0
Other education	5	1.7
Family income		
<18,750 SAR	116	38.8
>18,750 SAR	183	61.2

Table 2 provides insights into parents’ knowledge regarding the vaccination of their children in the study. The majority of parents (94.4%) agreed or strongly agreed that childhood vaccines protect their children from serious diseases. Similarly, a significant proportion (94.0%) agreed or strongly agreed that vaccinating their child is important for the health of others in their community. However, some misconceptions and concerns were observed. A notable finding was that 33.6% of parents agreed or strongly agreed that it is better for their child to acquire immunity by getting the disease rather than obtaining it through vaccination. Additionally, there were varying levels of agreement regarding the potential adverse effects of vaccines. For example, 26.6% of parents agreed or strongly agreed that vaccines can potentially cause autism, and 18.6% believed they could lead to learning difficulties.

**Table 2:** Knowledge of the parents regarding the vaccination of their children.

Items	Frequency	Percent (%)
Childhood vaccines protect my child from serious diseases		
Disagree or strongly disagree	7	2.3
Neutral	10	3.3
Agree or strongly agree	284	94.4
Vaccinating my child is important for the health of others in my community		
Disagree or strongly disagree	18	6.0
Agree or strongly agree	283	94.0
It is better for my child to acquire immunity by getting the disease rather than through vaccination		
Disagree or strongly disagree	200	66.4
Agree or strongly agree	101	33.6
Vaccines can potentially cause learning difficulties		
Disagree or strongly disagree	245	81.4
Agree or strongly agree	56	18.6
Vaccines can potentially cause autism		
Disagree or strongly disagree	221	73.4
Agree or strongly agree	80	26.6
Vaccines can potentially cause the following diabetes		
Disagree or strongly disagree	246	81.7
Agree or strongly agree	55	18.3
Vaccines can potentially cause the following sudden infant death		
Disagree or strongly disagree	242	80.4
Agree or strongly agree	59	19.6
Vaccines can potentially cause other chronic diseases		
Disagree or strongly disagree	222	73.8
Agree or strongly agree	79	26.2
Sufficient safety tests are not conducted for vaccines		
Disagree or strongly disagree	183	60.8
Agree or strongly agree	118	39.2
Vaccines are used for all age groups, not just for children		
Disagree or strongly disagree	20	6.6
Agree or strongly agree	281	93.4
The harm of vaccines outweighs their benefits		
Disagree or strongly disagree	233	77.4
Agree or strongly agree	68	22.6
Vaccines for children have globally unified principles and guidelines		
Disagree or strongly disagree	9	3.0
Agree or strongly agree	292	97.0
Do parents in Saudi Arabia receive sufficient information about vaccines and their safety?		
Disagree or strongly disagree	117	38.9
Agree or strongly agree	184	61.1
The optimal number of vaccines that I believe my child should receive in one doctor’s visit is		
1–2 vaccines	123	40.9
3–4 vaccines	67	22.3
>4 vaccines	14	4.7
Based on doctors’ recommendation	97	32.2

Table 3 provides insights into parents’ beliefs regarding the vaccination of their children. Half (51.8%) of the parents agreed or strongly agreed that new vaccines carry more risks than old vaccines, while 48.2% disagreed or strongly disagreed. Most (69.1%) agreed or strongly agreed with the objection to their child receiving more than five types of vaccines during a single doctor’s visit, while 30.9% disagreed or strongly disagreed. Regarding the belief that a child receiving a high number of vaccines during the first two years of life may weaken their immune system, 38.9% agreed or strongly agreed, while 61.1% disagreed or strongly disagreed. Regarding the perception of vaccines being given to immunize children against non-dangerous diseases, 45.8% agreed or strongly agreed, while 54.2% disagreed or strongly disagreed.

**Table 3:** Beliefs of the parents regarding the vaccination of their children.

Items	Frequency	Percent (%)
New vaccines carry more risks than old vaccines		
Disagree or strongly disagree	145	48.2
Agree or strongly agree	156	51.8
I do not object to my child receiving more than five types of vaccines during a single visit to the doctor		
Disagree or strongly disagree	93	30.9
Agree or strongly agree	208	69.1
My child receives a lot of vaccines, ranging from 10–15, during the first two years of his life, which may weaken his/her immune system		
Disagree or strongly disagree	184	61.1
Agree or strongly agree	117	38.9
Vaccines are given to children to immunize them against non-dangerous diseases		
Disagree or strongly disagree	163	54.2
Agree or strongly agree	138	45.8
Vaccines make the immune system stronger against diseases		
Disagree or strongly disagree	18	6.0
Agree or strongly agree	280	94.0
There is no need for vaccines against polio and measles due to the eradication of these diseases		
Disagree or strongly disagree	245	81.4
Agree or strongly agree	56	18.6
There are cases where the administration of vaccines containing live bacteria or viruses is prohibited, such as the polio vaccine, typhoid vaccine, and triple vaccine		
Disagree or strongly disagree	39	13.0
Agree or strongly agree	262	87.0
A child who is in good health does not need vaccines		
Disagree or strongly disagree	243	80.7
Agree or strongly agree	58	19.3

A majority of parents (94%) agreed or strongly agreed that vaccines make the immune system stronger against diseases, while only 6% disagreed or strongly disagreed. Additionally, the belief that vaccines against polio and measles are unnecessary due to their eradication was held by 18.6% of parents, with 81.4% disagreeing or strongly disagreeing with this statement. Concerning the belief about the prohibition of administering vaccines containing live bacteria or viruses, such as the polio vaccine, typhoid vaccine, and triple vaccine, 87% of parents agreed or strongly agreed, while 13% disagreed or strongly disagreed. Lastly, the notion that a child in good health does not need vaccines was disagreed or strongly disagreed with by 80.7% of parents, while 19.3% agreed or strongly agreed (Table 3).

Table 4 presents the parents’ confidence and attitudes toward the vaccination of their children. Regarding trust in the information received about vaccines, the majority of parents (80.4%) agreed or strongly agreed with it, while a small percentage (2.7%) disagreed or strongly disagreed. However, 16.9% remained neutral on this statement. Regarding hesitation in giving vaccines to their child, 25.2% of parents agreed or strongly agreed that they were often hesitant, while most (60.5%) disagreed or strongly disagreed, and 14.3% expressed neutrality on this issue. The majority (51.2%) reported never being hesitant about the topic of vaccines in general, while 23.6% indicated being hesitant sometimes, and smaller percentages (17.9% and 7.3%) mentioned being hesitant often or always, respectively (Table 4).

**Table 4:** Parents’ confidence in the vaccination of their children.

Items	Frequency	Percent (%)
I trust the information I receive about vaccines		
Disagree or strongly disagree	8	2.7
Neutral	51	16.9
Agree or strongly agree	242	80.4
Often, I am hesitant to give the vaccine to my child		
Disagree or strongly disagree	182	60.5
Neutral	43	14.3
Agree or strongly agree	76	25.2
Generally, I consider myself to be hesitant when it comes to the topic of vaccines		
Never	154	51.2
Sometimes	71	23.6
Often	54	17.9
Always	22	7.3
There is an alternative method to protect my child from diseases instead of vaccines, which is maintaining general hygiene and having a healthy diet		
Disagree or strongly disagree	220	73.1
Neutral	35	11.6
Agree or strongly agree	46	15.3
I am worried about the potential side effects that vaccines may cause		
Disagree or strongly disagree	87	28.9
Neutral	80	26.6
Agree or strongly agree	134	44.5
I am concerned about any new vaccine, fearing its potential side effects and its efficacy compared to the old vaccines due to limited testing and insufficient monitoring		
Disagree or strongly disagree	39	13.0
Neutral	82	27.2
Agree or strongly agree	180	59.8
I want to obtain more comprehensive information about vaccines to alleviate my concerns.		
Yes	237	78.7
I don't know	35	11.6
No	29	9.6

A small portion of parents (15.3%) agreed or strongly agreed that there is an alternative method to protect their child from diseases instead of vaccines, such as maintaining general hygiene and maintaining a healthy diet. However, the majority (73.1%) disagreed or strongly disagreed with this statement. Concerning worries about the potential side effects of vaccines, 44.5% agreed or strongly agreed, 26.6% remained neutral, and 28.9% disagreed or strongly disagreed. Similarly, when asked about concerns regarding new vaccines compared to old ones due to limited testing and insufficient monitoring, 59.8% agreed or strongly agreed with being concerned, 27.2% were neutral, and 13.0% disagreed or strongly disagreed. The desire for more comprehensive information about vaccines to alleviate concerns was expressed by the majority of parents (78.7%), while 6% indicated a lack of interest in obtaining more information, and 11.6% responded with uncertainty (Table 4).

Table 5 provides insights into parents’ attitudes and satisfaction toward the vaccination of their children in the study. The majority of parents (67.4%) reported always being in favor of vaccination, while smaller percentages indicated being in favor sometimes (4.3%), often (25.9%), or never (2.3%). When asked about knowing parents who did not vaccinate their children for religious, ideological, or cultural reasons, approximately one-third of parents (34.2%) agreed or strongly agreed, 40.2% disagreed or strongly disagreed, and 25.6% remained neutral on this statement. A significant majority (92.4%) agreed or strongly agreed, while a small proportion (2.7%) disagreed or strongly disagreed with adherence to pediatricians’ recommendations on vaccines, and 5.0% responded with neutrality. Concerning satisfaction with the vaccination program of the Ministry of Public Health, the majority of parents (85.7%) agreed or strongly agreed, while a smaller percentage (3.0%) disagreed or strongly disagreed, and 11.3% remained neutral. Regarding satisfaction with the answers received from the pediatrician regarding their child’s vaccines, the majority of parents (82.4%) agreed or strongly agreed, while 5.6% disagreed or strongly disagreed, and 12.0% responded with neutrality (Table 5).

**Table 5:** Parents’ attitudes and satisfaction toward vaccination of their children.

Items	Frequency	Percent (%)
I am in favor of vaccination		
Never	7	2.3
Sometimes	13	4.3
Often	78	25.9
Always	203	67.4
I know parents who do not vaccinate their children for religious, ideological, or cultural reasons		
Disagree or strongly disagree	121	40.2
Neutral	77	25.6
Agree or strongly agree	103	34.2
Usually, I adhere to the recommendations of my pediatricians regarding my child’s/children’s vaccines.		
Disagree or strongly disagree	8	2.7
Neutral	15	5.0
Agree or strongly agree	278	92.4
I am satisfied with the vaccination program of the Ministry of Public Health		
Disagree or strongly disagree	9	3.0
Neutral	34	11.3
Agree or strongly agree	258	85.7
I am satisfied with the way vaccines are administered when it is done by someone other than my child’s personal pediatrician (for example, a nurse, medical student, or resident doctor)		
Disagree or strongly disagree	53	17.6
Neutral	66	21.9
Agree or strongly agree	182	60.5
I am satisfied with the answers I receive from my pediatrician regarding my child’s/children’s vaccines.		
Disagree or strongly disagree	17	5.6
Neutral	36	12.0
Agree or strongly agree	248	82.4
If I get blessed with another child today, I want them to receive all the recommended vaccines		
Yes	263	87.4
I don’t know	27	9.0
No	11	3.7
I recommend others take vaccines		
Never	7	2.3
Sometimes	17	5.7
Often	77	25.8
Always	198	66.2

The majority of the participants (87.4%) indicated that if they have another child, they would want them to receive all the recommended vaccines. When asked about recommending others to take vaccines, the majority of parents (66.2%) reported always recommending, followed by those who often recommend (25.8%).

The majority of parents (80.7%) reported that their children had received all the vaccines recommended by their pediatrician. A smaller proportion (18.3%) stated that their child had received only mandatory vaccines, while 1% responded with “I don’t know.” The majority (83.1%) reported not refusing or delaying vaccinating their child. More than half of the parents (54.5%) indicated that their child had received a rotavirus vaccine. Approximately one-third of parents (35.2%) stated that they have vaccinated or will vaccinate their child against cervical cancer or genital warts. A similar percentage (35.9%) responded with “I don’t know,” and 28.9% reported that they had not vaccinated their child against cervical cancer or genital warts (Table 6).

**Table 6:** Practices of the parents regarding the vaccination of their children.

Items	Frequency	Percent (%)
My child has received the vaccinations recommended by their pediatrician		
Yes, all vaccines	243	80.7
Yes, only mandatory vaccines	55	18.3
I don’t know	3	1.0
I had to refuse or delay vaccinating my child in the past		
Yes	31	10.3
I don’t know	20	6.6
No	250	83.1
My child has received the rotavirus vaccine		
Yes	164	54.5
I don’t know	89	29.6
No	48	15.9
I have vaccinated or will vaccinate my child against cervical cancer or genital warts		
Yes	106	35.2
I don’t know	108	35.9
No	87	28.9

Table 7 presents the association between respondents’ characteristics and their knowledge about their children’s vaccination. Mothers were more likely to disagree or strongly disagree with the statement that a child in good health does not need vaccines than fathers (87.5%) (p = 0.042). Respondents with university degrees were also more likely to disagree with the statement (p = 0.017). Regarding nationality, Saudi respondents were more likely to disagree with a statement (p = 0.001) (Table 7). Parents with a first child were also more likely to disagree than those with more children (p = 0.013).

**Table 7:** Association between respondents’ characteristics and knowledge about their children’s vaccination. *: Statistically significant.

Characteristics	A child who is in good health does not need vaccines		Chi-square	P-value
	Disagree or strongly disagree	Agree or strongly agree		
Gender of the parent				
Mother	159	46	4.1	0.042*
	77.6%	22.4%		
Father	84	12		
	87.5%	12.5%		
Parent’s educational level				
Secondary school	13	9	8.154	0.017*
	59.1%	40.9%		
University degree	225	49		
	82.1%	17.9%		
Other	5	0		
	100.0%	0.0%		
Nationality				
Saudi	241	47	37.3	0.001*
	83.7%	16.3%		
Non-Saudi	2	11		
	15.4%	84.6%		
Child order				
The first child	160	28	6.16	0.013*
	85.1%	14.9%		
Other children	83	30		
	73.5%	26.5%		
Family income				
Low income	87	29	4.9	0.27
	75.0%	25.0%		
High income	156	27		
	85.2%	14.8%		

Table 8 shows the association between respondents’ characteristics and hesitancy to vaccinate their children. There was an association between vaccine hesitancy, the child order (p = 0.022), and education level (p = 0.028).

**Table 8:** Association between respondents’ characteristics and hesitancy to vaccinate their children. *: Statistically significant.

Characteristics	Often, I hesitate to give the vaccine to my child			Chi-square	P-value
	Disagree or strongly disagree	Neutral	Agree or strongly agree		
Gender of the parent					
Mother	123	27	55	1.2	0.544
	60.0%	13.2%	26.8%		
Father	59	16	21		
	61.5%	16.7%	21.9%		
Parent’s educational level					
Secondary school	13	7	2	10.9	0.028*
	59.1%	31.8%	9.1%		
University degree	164	36	74		
	59.9%	13.1%	27.0%		
Other education	5	0	0		
	100.0%	0.0%	0.0%		
Nationality					
Saudi	175	41	72	0.27	0.872
	60.8%	14.2%	25.0%		
Non-Saudi	7	2	4		
	53.8%	15.4%	30.8%		
Child order					
The first child	124	26	38	7.6	0.022*
	66.0%	13.8%	20.2%		
Other children	58	17	38		
	51.3%	15.0%	33.6%		
Family income					
Low income	62	22	32	4.6	0.103
	53.4%	19.0%	27.6%		
High income	118	21	44		
	64.5%	11.5%	24.0%		

## Discussion

Vaccination is a crucial step in preventing the spread of infectious diseases. Despite the well-established benefits of vaccination, vaccine hesitancy and refusal are growing concerns worldwide [13,14]. As parents play a significant role in deciding to vaccinate their children, evaluating their knowledge, beliefs, and attitudes toward vaccination is essential. This study aimed to estimate the rate of vaccination hesitancy and the level of knowledge and beliefs among parents regarding child vaccination. The study’s main outcomes include the prevalence of misconceptions about vaccinations and the knowledge level of parents regarding vaccination. The findings of this study can help identify the factors that contribute to vaccine hesitancy among parents and inform public health interventions aimed at promoting vaccination.

The results of this study showed that while most parents have a positive attitude toward childhood vaccination and recognize its benefits, there are still misconceptions and concerns surrounding vaccines. These findings are consistent with previous studies that have also identified vaccine hesitancy as a growing concern among parents. For instance, a study conducted by Larson et al. [9] found that vaccine hesitancy is prevalent worldwide, with varying levels of concern across different countries. Similarly, a study by Dubé et al. [15] identified factors contributing to vaccine hesitancy, such as individual beliefs and values, lack of trust in healthcare providers, and misinformation through social media. Moreover, another study also reported varying levels of vaccine confidence among parents, with some expressing concerns about vaccine safety and effectiveness [16]. This study adds to the growing evidence of vaccine hesitancy among parents. While the majority of parents in this study had a positive attitude toward vaccination, addressing misconceptions and concerns is crucial in promoting vaccine uptake and ensuring the health of children and the wider community.

The results of this study demonstrated a relatively high level of awareness and understanding among parents regarding the benefits of childhood vaccination. These results are consistent with previous studies on parental attitudes toward vaccination. For example, a review by Smith et al. [17] found that most parents agreed that vaccines are important for their child’s health. Similarly, another study aimed to assess parents’ attitudes toward the human papillomavirus vaccine found that most parents recognized the importance of vaccination in preventing infectious diseases [18]. However, similar to this study, other researchers have also found that misconceptions and concerns about vaccines remain prevalent among some parents. Research has shown that a significant proportion of parents have concerns about the safety and effectiveness of vaccines [19], with some still believing that vaccines can cause autism. Even in developed countries, research has found that vaccine hesitancy remains a concern in Canada, despite high levels of vaccine acceptance [20]. In a study conducted in 2010 by Chang et al., the rate of people agreeing that vaccines cause autism was higher among hesitant parents (p < 0.001) [21]. The belief that it is better for children to build immunity through natural infection has decreased from a quarter of the parents in a previous study [22] to 11.7% in this study. It is concerning that despite the overwhelming evidence that vaccines are safe and effective, some parents continue to hold misconceptions and hesitations toward vaccination. These beliefs can impact vaccination uptake, leading to outbreaks of vaccine-preventable diseases. Therefore, healthcare providers must be aware of these beliefs and work to educate and address any concerns, emphasizing the importance of vaccination. This study revealed that 51.8% of parents believed that new vaccines carry more risks than old vaccines, which is similar to findings reported by previous studies [23]. Moreover, 69.1% of parents objected to their child receiving more than five types of vaccines during a single doctor’s visit, which is higher than the rate reported in a previous study [24]. In contrast, the study found that 61.1% of parents disagreed that a high number of vaccines during the first two years of life might weaken their child’s immune system, which aligns with previous findings [25]. Besides, 54.2% of parents disagreed that vaccines are given against non-dangerous diseases, a belief previously reported in different populations [26,27].

Results indicated that most parents (94.0%) agreed that vaccines strengthen the immune system against diseases, which is consistent with previous studies [19]. Only 18.6% of parents believed that vaccines against polio and measles are unnecessary due to eradication, while 81.4% disagreed. This belief has also been previously reported in other studies [19]. Regarding the belief about the prohibition of administering vaccines containing live bacteria or viruses, 87.0% of parents agreed or strongly agreed, while 13.0% disagreed. This belief aligns with previous studies [19,28]. Importantly, the study found that 80.7% of parents disagreed that a child in good health does not need vaccines, while only 19.3% agreed. This finding supports previous research that indicates parental attitudes toward vaccines are generally positive.

Regarding the belief that a high number of vaccines during the first two years of life can weaken a child’s immune system, 61.1% of parents disagreed or strongly disagreed. This finding is consistent with previous studies that showed that this belief was more prevalent among vaccine-hesitant parents and those who refused vaccination [29].

Our findings indicated that the majority of parents (80.4%) agreed or strongly agreed with the trustworthiness of the information given about vaccines. This finding is consistent with previous studies [30,31], indicating that parents trust the information received. However, some (16.9%) remained neutral on this statement. This finding is similar to a study by Henrikson et al. [32], which reported that many parents were undecided regarding the trustworthiness of vaccine information. Regarding vaccine hesitancy, our study found that 25.2% of parents agreed or strongly agreed that they were often hesitant to give vaccines to their children. This result is supported by a study conducted by Opel et al. [31], which found that approximately one-third of parents reported vaccine hesitancy.

Regarding general vaccine hesitancy, the majority (51.2%) of parents reported never being hesitant, while 23.6% indicated being hesitant sometimes. This finding is in line with a study conducted by Larson et al. [9], which found that only a small proportion of parents (8%) remained consistently vaccine-hesitant over several years. The findings of our study showed that 15.3% of parents agreed or strongly agreed that maintaining general hygiene and having a healthy diet is an alternative method to protect their child from diseases instead of vaccines. Similarly, previous studies found that a significant number of parents believe that boosting their child’s immune system through natural means is a safer alternative to vaccines [22,33].

Concerning worries about the potential side effects of vaccines, our study found that 44.5% of parents agreed with this statement. This finding is in line with another study, which found that many parents have concerns about vaccine safety [19]. The desire for more comprehensive information about vaccines to alleviate concerns was expressed by the majority of parents (78.7%) in our study. This finding is supported by previous studies which have suggested that effective communication between healthcare providers and parents is crucial in addressing vaccine hesitancy [33,34]. Regarding parents’ attitudes, the results revealed that most parents always favor vaccination for their children (67.4%). This finding aligns with previous research conducted in Saudi Arabia that reported favorable attitudes toward vaccination among parents [35]. However, it is important to note that a small percentage of parents reported favoring vaccination sometimes (4.3%) or often (25.9%), indicating some hesitancy toward vaccination. Regarding reasons why parents may not vaccinate their children, opinions varied, with approximately one-third of parents (34.2%) agreeing that they know parents who do not vaccinate their children for religious, ideological, or cultural reasons. This result may suggest that certain groups in Saudi Arabia may have unique concerns about vaccination, as noted in previous studies [36]. However, it is noteworthy that a significant proportion of parents disagreed with this statement (40.2%), suggesting that religious, ideological, or cultural concerns may not be the primary drivers of vaccine hesitancy among Saudi parents.

Regarding adherence to pediatricians’ recommendations on vaccines, a high proportion of parents (92.4%) agreed or strongly agreed with vaccinating their children in accordance with their pediatrician’s recommendations. This finding is consistent with previous research in Saudi Arabia, which highlighted the role of healthcare providers in increasing vaccine uptake among parents [37,38]. However, it is essential to note that few parents still expressed disagreement or neutrality toward following pediatricians’ vaccination recommendations (2.7% and 5.0%, respectively), indicating that healthcare providers may need to address these concerns among vaccine-hesitant parents.

The study results revealed that a majority of parents were satisfied with the vaccination program of the Ministry of Public Health (85.7%). This finding is consistent with earlier research that reported high satisfaction levels with the Saudi Arabian healthcare system [8]. However, approximately 11.3% of parents remained neutral, suggesting that there may be areas for improvement in the Saudi Arabian vaccination program. The responses were diverse regarding satisfaction with the way vaccines are administered by someone other than the child’s personal pediatrician. Previous studies noted that trust in healthcare providers is essential for vaccine uptake among parents [8]. However, this study revealed that only 59.4% of parents agreed or strongly agreed that they were satisfied with how vaccines are administered by someone other than their child’s personal pediatrician. This finding emphasizes the importance of building trust in healthcare providers outside of the child’s personal pediatrician to increase vaccine uptake.

We found that when it comes to vaccination against cervical cancer or genital warts, only approximately one-third of parents (35.2%) stated that they have vaccinated or will vaccinate their child, which is lower than the findings of studies conducted in other countries [39]. A similar percentage (35.9%) responded with “I don’t know,” indicating a lack of knowledge or awareness about the vaccine. This is consistent with previous studies that reported low knowledge and awareness about the human papillomavirus vaccine among parents [40]. Nearly 30% of parents reported not vaccinating their children against cervical cancer or genital warts. This is concerning as the human papillomavirus vaccine is safe and effective in preventing cervical cancer and other human papillomavirus-related diseases. Low vaccination rates for human papillomavirus may be due to various reasons, such as lack of awareness, perceived risk, and misinformation.

Regarding refusing or delaying vaccination, a small proportion of parents confirmed that they had to refuse (10.3%), while the majority reported that they had not refused or delayed vaccinating their child (83.1%). These results are consistent with previous studies that reported low vaccine refusal and delay levels among parents [38]. Regarding the rotavirus vaccine, more than half of the parents (54.5%) indicated that their child had received it. However, nearly one-third (29.6%) reported not knowing if their child had received the vaccine. This highlights the need for improved communication and education about the rotavirus vaccine’s importance, safety, and efficacy. The results indicated that there was no statistically significant association between parents’ gender and vaccine hesitancy, with both mothers and fathers having similar distributions across the response categories. This finding is consistent with previous studies that have reported no gender-based differences in vaccine hesitancy [41,42]. However, the study found a statistically significant association between educational level and vaccine hesitancy. Respondents with a secondary school education showed a higher percentage of vaccine hesitancy than those with a university degree. This result aligns with previous studies that reported a negative correlation between educational level and vaccine hesitancy [43].

The findings of this study contribute to the understanding of factors associated with vaccine hesitancy and highlight the importance of education in promoting vaccine acceptance. Healthcare providers should help improve health literacy and provide accurate information to hesitant parents to help them make informed decisions about childhood vaccinations.

The results revealed that respondents with a first child had higher vaccine hesitancy than those with other children. This finding is consistent with previous research that has reported that first-time parents are more likely to be vaccine-hesitant than parents with more than one child [44]. Thus, there is a need for targeted strategies to address vaccine hesitancy among first-time parents. Although the association between income and hesitancy was not statistically significant, previous studies have reported mixed results regarding the association between family income and vaccine hesitancy.

This study has some limitations that should be considered when interpreting the results. First, the study relied on self-reported data, which may be susceptible to response bias, as parents may have provided socially desirable responses rather than their true beliefs and attitudes. This study had a 22% non-response rate which might be attributed to the questionnaire with many questions as it had 50 questions that may have caused respondents not to complete it. However, the number of responses (301) received stayed above the minimum sample size calculated (300 participants) to give accurate results. Furthermore, the study focused mainly on the KAP of parents regarding childhood vaccination but did not explore the underlying reasons. Hence, qualitative studies aimed at exploring reasons are recommended. This study did not assess the association between vaccine hesitancy among parents and the actual vaccination status of their children. Therefore, it is unclear if vaccine hesitancy observed in this study translates into lower vaccination rates. Further research is needed on the association between parental attitudes and vaccination behavior among children.

## Conclusions

This study assessing parents’ KAP regarding children’s vaccination revealed a lack of consensus among parents. While some parents held misconceptions about the risks associated with new vaccines and preferred natural immunity over vaccination, the majority expressed concerns about the potential adverse effects of vaccines but recognized their effectiveness in strengthening the immune system. Some parents were hesitant about vaccinating their children and believed in alternative methods of disease prevention. Therefore, providing comprehensive information to address these concerns is crucial. Despite the varying opinions, the study found that the majority of parents were in favor of vaccinating their children. They followed pediatricians’ recommendations, expressed satisfaction with the vaccination program, and were willing to have their future children receive all recommended vaccines. Gender, educational level, and nationality influenced parents’ KAP regarding vaccination, highlighting the importance of tailored communication and education efforts. Education level was also associated with hesitancy. Therefore, improving understanding and addressing concerns through targeted interventions, including education, can contribute to higher vaccination rates and better overall health outcomes for children.
